# Oral supplementation of healthy adults with 2′-*O*-fucosyllactose and lacto-*N*-neotetraose is well tolerated and shifts the intestinal microbiota

**DOI:** 10.1017/S0007114516003354

**Published:** 2016-10-10

**Authors:** Emma Elison, Louise K. Vigsnaes, Laura Rindom Krogsgaard, Julie Rasmussen, Nikolaj Sørensen, Bruce McConnell, Thierry Hennet, Morten O. A. Sommer, Peter Bytzer

**Affiliations:** 1 Glycom A/S, Diplomvej 373, DK-2800 Kgs. Lyngby, Denmark; 2 Department of Medicine, Zealand University Hospital, Lykkebækvej 1, DK-4600 Køge, Denmark; 3 Department of Clinical Medicine, Copenhagen University, DK-2200 Copenhagen, Denmark; 4 Clinical-Microbiomics ApS, Kogle Allé 5, DK-2970 Hørsholm, Denmark; 5 Institute of Physiology and Zurich Centre of Integrative Human Physiology, University of Zurich, Zurich CH-8057, Switzerland; 6 Novo Nordisk Foundation Center for Biosustainability, Technical University of Denmark, Kogle Alle 6, DK-2970 Hørsholm, Denmark

**Keywords:** 2′-*O*-Fucosyllactose, Lacto-*N*-neotetraose, Clinical study, Safety, Tolerance

## Abstract

The gut microbiota has been established as an important player influencing many aspects of human physiology. Breast milk, the first diet for an infant, contains human milk oligosaccharides (HMO) that shape the infant’s gut microbiota by selectively stimulating the growth of specific bacteria, especially bifidobacteria. In addition to their bifidogenic activity, the ability of HMO to modulate immune function and the gut barrier makes them prime candidates to restore a beneficial microbiota in dysbiotic adults and provide health benefits. We conducted a parallel, double-blind, randomised, placebo-controlled, HMO-supplementation study in 100 healthy, adult volunteers, consuming chemically produced 2′-*O*-fucosyllactose (2′FL) and/or lacto-*N*-neotetraose (LNnT) at various daily doses and mixes or placebo for 2 weeks. All participants completed the study without premature discontinuation. Supplementation of 2′FL and LNnT at daily doses up to 20 g was shown to be safe and well tolerated, as assessed using the gastrointestinal symptoms rating scale. 16S rRNA sequencing analysis showed that HMO supplementation specifically modified the adult gut microbiota with the primary impact being substantial increases in relative abundance of Actinobacteria and *Bifidobacterium* in particular and a reduction in relative abundance of Firmicutes and Proteobacteria. This study provides the first set of data on safety, tolerance and impact of HMO on the adult gut microbiota. Collectively, the results from this study show that supplementing the diet with HMO is a valuable strategy to shape the human gut microbiota and specifically promote the growth of beneficial bifidobacteria.

Intensive research over the past decade has revealed the gut microbiota to be an important player in host health by influencing many aspects of human physiology, including energy metabolism^(^ ^1^ ^)^, hormonal balance^(^ ^2^ ^)^ and immunity^(^ ^3^ ^,^ ^4^ ^)^. The gut microbiota also contributes to establishment of the mucosal barrier and maintenance of intestinal homoeostasis^(^ ^5^ ^)^. The microbiota of the human intestine is a complex and very dynamic microbial ecosystem, and extensive research has been able to link imbalance in the intestinal bacterial population to a wide variety of both intestinal and extra-intestinal diseases such as malnutrition, cancer, inflammatory diseases, metabolic diseases, gastrointestinal (GI) diseases and response to pathogens^(^ ^6^ ^–^ ^14^ ^)^. This research has led to an increasing appreciation of the gut microbiota as a target for therapeutic intervention. Indeed, modulation of the gut microbiota has been shown to be a promising therapeutic approach to treat recurrent *Clostridium difficile* infections^(^ ^15^ ^)^.

Breast milk, the first diet for an infant, offers all the essential nutrients for growth and development, and provides bioactive factors such as Ig, antimicrobial proteins and cytokines^(^ ^16^ ^)^. In addition, components of breast milk are able to shape the intestinal microbiota and drive the maturation of the infant gut. The major components driving this are the human milk oligosaccharides (HMO), as can be seen when comparing breast-fed with formula-fed infants, where breast-fed infants carry a more stable and uniform microbial population than formula-fed infants^(^ ^17^ ^)^. HMO are a family of highly diverse structures of unconjugated glycans, present in high concentrations in human milk. The structural diversity they represent can be broadly divided into fucosylated, sialylated and non-fucosylated neutral structures. One litre of mother’s milk contains 5–25 g of HMO^(^ ^18^ ^)^, and HMO therefore are the third most abundant solid constituent in human milk. HMO are not digested in the upper GI tract, and only 1–2 % is absorbed in infants^(^ ^19^ ^,^ ^20^ ^)^. The majority of ingested HMO reach the large intestine where they provide selective substrates for specific gut bacteria^(^ ^21^ ^–^ ^27^ ^)^, modulate the immune system^(^ ^28^ ^–^ ^31^ ^)^ and prevent the epithelial adhesion of intestinal pathogens^(^ ^32^ ^–^ ^36^ ^)^.

After weaning, the introduction of solid food profoundly influences the microbial ecology. In fact, diet is, together with genetics and environmental factors, one of the main contributors to the diversity of human intestinal microbiota^(^ ^37^ ^)^. Dietary manipulation hence represents a strategy to promote a beneficial GI microbial community and to improve the well-being of the host.

Selective stimulation of beneficial intestinal bacteria by promoting their growth and metabolic activity may be a helpful approach in creating a beneficial microbial community. As some bacteria are able to produce a large set of carbohydrate active enzymes, including glycoside-hydrolases and transporters, they can grow on carbon sources, which are unfermented by other members of the intestinal microbial community. HMO are probably best known for their prebiotic effects in breast-fed infants, where they exert a strong bifidogenic effect, characterised by the proliferation of specific strains including *Bifidobacterium infantis*, *B. breve* and *B. bifidum* ^(^ ^17^ ^,^ ^38^ ^)^. Bifidobacteria are generally considered beneficial for human health because of their ability to digest complex carbohydrates and dietary fibres. Further, low bifidobacteria abundance has been linked to GI^(^ ^39^ ^,^ ^40^ ^)^ and metabolic diseases^(^ ^41^ ^,^ ^42^ ^)^, for example. As *Bifidobacterium* is highly abundant in the microbiota of breast-fed infants, their acquisition and HMO metabolism have drawn a lot of attention in recent years^(^ ^43^ ^)^. *In vitro* fermentation studies have clearly confirmed the decisive role of HMO in promoting the growth of bifidobacteria^(^ ^25^ ^)^. However, the impact of HMO on the adult intestinal microbiota and adult GI tract is unknown. We designed a prospective study to assess the effects of HMO supplementation on the composition of the adult gut microbiota and on GI symptoms. We selected 2′-*O*-fucosyllactose (2′FL) as a fucosylated HMO and lacto-*N*-neotetraose (LNnT) as a non-fucosylated neutral HMO. These two compounds are among the shortest HMO to remain unaltered after passage through the small intestine and are available for clinical use. This first human study of HMO supplementation in adults provided valuable insights into the effect of HMO on the adult gut microbiota. In addition, the study assessed the safety and tolerability of HMO supplementation in adults.

## Methods

### Subjects

Subjects were recruited from the region Zealand in Denmark. In total, 110 healthy male and female adult volunteers were invited for screening. From this pool of volunteers, 100 subjects were randomised to participate in the study. Inclusion criteria were as follows: aged between 18 and 60 years, ability and willingness to understand and comply with the study procedures and sign the written informed consent. Exclusion criteria were as follows: participation in a clinical study 1 month before the screening visit and throughout the study, abnormal results of screening laboratory and clinical tests relevant for study participation, any GI symptom scoring >3 on the Gastrointestinal Symptom Rating Scale (GSRS), a mean score on the total GSRS>2 during the screening period, any GI and/or other severe diseases, highly dosed probiotic supplement and/or antibiotic use 3 months before the study and throughout the study, consumption on a regular basis of medication that might interfere with symptom evaluation, pregnancy or seeking pregnancy and nursing. A summary of the trial design was registered at www.ClinicalTrials.gov (NCT01927900).

### Study products

All carbohydrate compounds were provided as powder in PET bottles. HMO 2′FL and LNnT were supplied by Glycom A/S as white, free-flowing, crystalline powders of synthetic origin at 99·9 % (2′FL) and 98·9 % (LNnT) purity, respectively. The samples were subjected to preclinical toxicology studies^(^ ^44^ ^,^ ^45^ ^)^. Furthermore, an European Food Safety Authority (EFSA) panel on Dietetic Products, Nutrition and Allergies (NDA) specifically assessed 2′FL and LNnT and concluded that these, as produced by Glycom A/S, are safe to use in foods^(^ ^46^ ^,^ ^47^ ^)^. Glucose (Dextropure; Valora Trade Denmark A/S) was given as placebo. Subjects were asked to dissolve the contents of the bottles immediately before consumption by mixing the powder with water, and were asked to consume the product every day at breakfast.

### Study design

The present study was a parallel, double-blind, randomised, placebo-controlled, dose-finding study. After a screening visit and a run-in period of 1–2 weeks, eligible volunteers were randomly assigned by a computer-generated list to ten groups of ten participants each, consuming either HMO or placebo daily for 2 weeks. A constant regimen of 2′FL, LNnT or 2′FL+LNnT (2:1 mass ratio; mix) at 5, 10 or 20 g per d or 2 g of glucose as placebo was allocated to each group. The daily doses were chosen to be within the range of the average daily intake per kg body weight in infants^(^ ^46^ ^,^ ^47^ ^)^. Diet was not controlled, but subjects were asked not to change their diet over the course of the study. Subjects had clinical check-ups at entry and at the end of the intervention. Subjects taking the study product for ≥12 of the 14 d of intervention were considered compliant.

### Ethical considerations

This study was conducted according to the guidelines laid down in the Declaration of Helsinki, and all procedures involving human subjects were approved by the Ethics Committee in Region Zealand (registration number SJ-345). The trial was registered with the Danish Data Protection authorities via the regional approval system, and Danish regulations relating to personal data protection were respected. All subjects were given oral and written information about the purpose and procedures of the study. Consent to participate was signed by the subjects before the study started, and the subjects were free to withdraw from the study at any time point without giving any explanation.

### Gastrointestinal symptoms and stool consistency

To evaluate the influence on GI symptoms, participants completed a self-administered GSRS form, once at screening, once at entry and once at end of the intervention period. The GSRS form includes fifteen items covering five dimensions: abdominal pain, indigestion, reflux, diarrhoea and constipation^(^ ^48^ ^)^. The participants rated severity using a seven-point Likert scale running from (1) no discomfort to (7) very severe discomfort. Bowel movement frequency was recorded daily, and stool consistency was evaluated using the Bristol Stool Form Scale (BSFS)^(^ ^49^ ^)^. The BSFS was filled in on a daily basis during the study period, from screening to the end of the intervention. Adverse events, defined as any untoward medical occurrence, including those that did not necessarily have a causal relationship with the investigational or placebo products, were reported from intake of the first dose and throughout the intervention period.

### Blood analysis

Blood samples for routine clinical chemistry and haematology analyses were collected at screening and at the end of the intervention to assess the safety of study product intake. Samples were analysed for Hb, erythrocytes, haematocrit, leucocytes, thrombocytes, creatinine, Na, K, alanine aminotransferase, alkaline phosphatases, coagulation factor II, VII and X, bilirubin, albumin, C-reactive protein and glucose. Blood samples for the analysis of additional biomarkers were collected at study entry and at the end of the intervention. These samples were analysed for HbA1c, apoA1, apoB, transferrin, progesterone, cortisol, oestradiol, IL-10, IL-6, TNF-*α*, blood urea nitrogen, Fe, TAG, HDL-cholesterol, LDL-cholesterol, total free fatty acids, insulin, lysozyme, testosterone and glucagon (Unilabs A/S).

### Faecal biomarkers

Faecal samples for biomarker analysis were collected just before study entry and at the end of the intervention. ELISA was applied to determine calprotectin (Bühlmann Laboratories) and secretory IgA levels (Bethyl Laboratories). SCFA were analysed as described previously^(^ ^32^ ^)^. In brief, faecal samples were solubilised in 5 volumes of water, spiked with 10 mm-succinic acid and extracted twice in diethylether for 20 min at room temperature. The final supernatants were passed through a 0·45-μm filter and analysed by HPLC. The HPLC system (Lachrom L7100; Merck-Hitachi) included a HPX-87H Aminex column (300×7·8 mm, from BioRad) and guard column of the same type. Chromatography was performed at 30°C isocratically in 10-mm-H_2_SO_4_, which was the mobile phase, at a flow rate of 0·4 ml/min. SCFA were detected at 210 nm in a UV detector, and concentrations calculated from the peak areas were compared with authentic standards.

### Faecal DNA preparation

Faecal microbiota composition was analysed using four faecal samples per subject, with approximately 1 week between each sampling. Samples 1 and 2 were collected before intervention start, and samples 3 and 4 were collected during the intervention. For the analysis, the average of samples 1 and 2 was used as the baseline value and the average of samples 3 and 4 was for the intervention. The faecal samples were collected by the participants, and immediately stored in a freezer (about −20°C). When delivered to the hospital (in cooling kits), samples were stored at −80°C until analysis. DNA was extracted using the ninety-six-well PowerSoil DNA Isolation Kit (MO-BIO).

### 16S rRNA sequencing

The V3–V4 region of the 16S rDNA was amplified using the forward primer S-D-Bact-0341-b-S-17 (5′-TCGTCGGCAGCGTCAGATGTGTATAAGAGACAG-3′) and the reverse primer S-D-Bact-0785-a-A-21 (5′-GTCTCGTGGGCTCGGAGATGTGTATAAGAGACAG-3′)^(^ ^50^ ^)^, with Illumina adapters attached. The following PCR programme was used: 98°C for 30 s, 25× (98°C for 10 s, 55°C for 20 s, 72°C for 20 s) and 72°C for 5 min; Nextera Index Kit V2 (Illumina) indices were added in an identical PCR with only eight cycles. Products from the PCR reactions were cleaned using the SequalPrep Normalization Plate Kit (Invitrogen) or Agencourt AMPure XP PCR purification kit (Beckman Coulter) and pooled. Sequencing was carried out on an Illumina MiSeq sequencer using the MiSeq Reagent Kit V3 (Illumina) for 2× 300-bp paired-end sequencing.

### Bioinformatical analysis

The paired-end reads were merged, and low-quality sequences were discarded (truncating reads at a quality score of 4 or less and requiring 100-bp overlap between paired reads, perfect match to primers, a merged sequence length of 300–600 bp, a maximum of five expected errors and a minimum of 5 identical sequences in the data set). Sequences were clustered into operational taxonomic units (OTU) at 97 % sequence similarity using USEARCH^(^ ^51^ ^)^, and suspected chimeric sequences were discarded on the basis of UCHIME^(^ ^52^ ^)^. Taxonomic assignment of OTU was performed on the basis of comparison with a database of curated sequences derived from the Ribosomal Database Project^(^ ^53^ ^)^. Samples were rarified to the lowest sequence number found in a sample (11 568 sequences). Both negative controls and mock communities were included in the analysis as quality controls.

### Statistical analysis

All participants completed the study as per protocol, and were included in the statistical analysis. Differences in GSRS scores, BSFS scores, biomarker and clinical chemistry, and haematology were analysed.

Analysis of difference between baseline and end of intervention in the microbiota profile, within each intervention group, was performed using the Mann–Whitney *U* tests using Bonferroni’s correction for multiple hypothesis testing.

Generalised UniFrac distances^(^ ^54^ ^)^ between samples were calculated at *α*=0·5 and with phyla abundance overlaid on the plots.

Change in sequence abundance (delta (end−entry)) was calculated, and a one-way ANOVA with Fisher’s LSD as *post hoc* test was used to compare the statistical difference in the change in Actinobacteria and bifidobacteria, and the three most dominant OTU identified within the *Bifidobacterium* genus comparing placebo with each intervention group. Difference in SCFA concentration at entry and end of intervention was analysed using a two-way repeated measures ANOVA. Non-parametric tests were used when data were not normally distributed as tested using the Shapiro–Wilk normality test. Multiple comparisons using Pearson’s correlation coefficient were calculated for SCFA and bifidobacteria, testing separately the groups taking 5, 10 or 20 g of HMO using Prism 6 (GraphPad Prism, version 6.05). In all cases, statistically significant differences were established at *P*<0·05.

## Results

### Safety and tolerance of 2′-*O*-fucosyllactose and lacto-*N*-neotetraose supplementation

In total, 100 healthy, adult volunteers (forty-nine females and fifty-one males) aged 19–57 years were enrolled in the study. Demographic parameters such as age, sex and BMI did not differ between groups at entry (Table 1). All subjects were examined physically at screening and at end of intervention. No change in clinical significance in any physical parameter including pulse rate and blood pressure was found during the 2-week intervention. To further assess the safety of HMO supplementation, blood samples were collected before and after intervention for routine clinical chemistry and haematology analyses. These analyses revealed no irregularities considered due to the intake of study products in any intervention group (online Supplementary Table S1), thus confirming the safety of the tested compounds.

**Table 1:** Participant demographics at entry (Mean values and standard deviations; means and ranges)

	2′FL						LNnT						Mix							
	20 g		10 g		5 g		20 g		10 g		5 g		20 g		10 g		5 g		Placebo	
	Mean	sd	Mean	sd	Mean	sd	Mean	sd	Mean	sd	Mean	sd	Mean	sd	Mean	sd	Mean	sd	Mean	sd
BMI (kg/m^2^)	27·8	7·4	24·7	1·9	25·8	4·2	24·9	4·9	25·0	5·5	24·8	4·4	22·4	1·8	26·5	3·8	24·8	3·6	26·8	6·3
Age (years)																				
Mean	39·9		33·4		38·3		39·0		34·8		34·6		29·3		37·1		38·9		34·9	
Range	25–55		21–51		27–52		22–57		23–51		26–47		19–45		19–56		23–53		25–47	
Males/females	4/6		5/5		4/6		4/6		4/6		9/1		8/2		4/6		6/4		3/7	

2′FL, 2′-*O*-fucosyllactose; LNnT, lacto-*N*-neotetraose; Mix, 2′FL:LNnT (2:1).

**Fig. 1:**
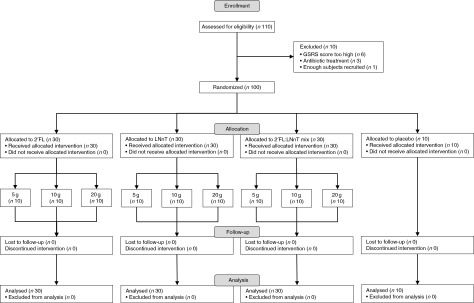
Flow chart of the study. A total of 110 healthy, adult volunteers were screened for eligibility to participate in the study; 100 of them were randomised to one of the following intervention groups: 2′-*O*-fucosyllactose (2′FL), lacto-*N*-neotetraose (LNnT) or 2:1 mix of 2′FL:LNnT, each in three daily doses of 5, 10 or 20 g, or 2 g glucose as placebo. GSRS, gastrointestinal symptom rating scale.

Compliance was defined as ingestion of the study product for ≥12 days during the intervention period. All subjects were compliant and completed the study according to the protocol without any dropouts (Fig. 1). A total of fifty-six adverse events were reported by forty-four subjects. All were judged as ‘mild’, and all subjects tolerated the investigational products throughout the trial period. Adverse events were usually reported as a complex of multiple symptoms such as flatulence, bloating and constipation, and were primarily reported at the end of the 2-week intervention. Most adverse events were reported by subjects taking the highest doses of 2′FL and LNnT. Gas/flatulence was the most common adverse event reported, followed by stomach pain, diarrhoea/loose stools and rumbling, but at lower frequencies.

**Fig. 2:**
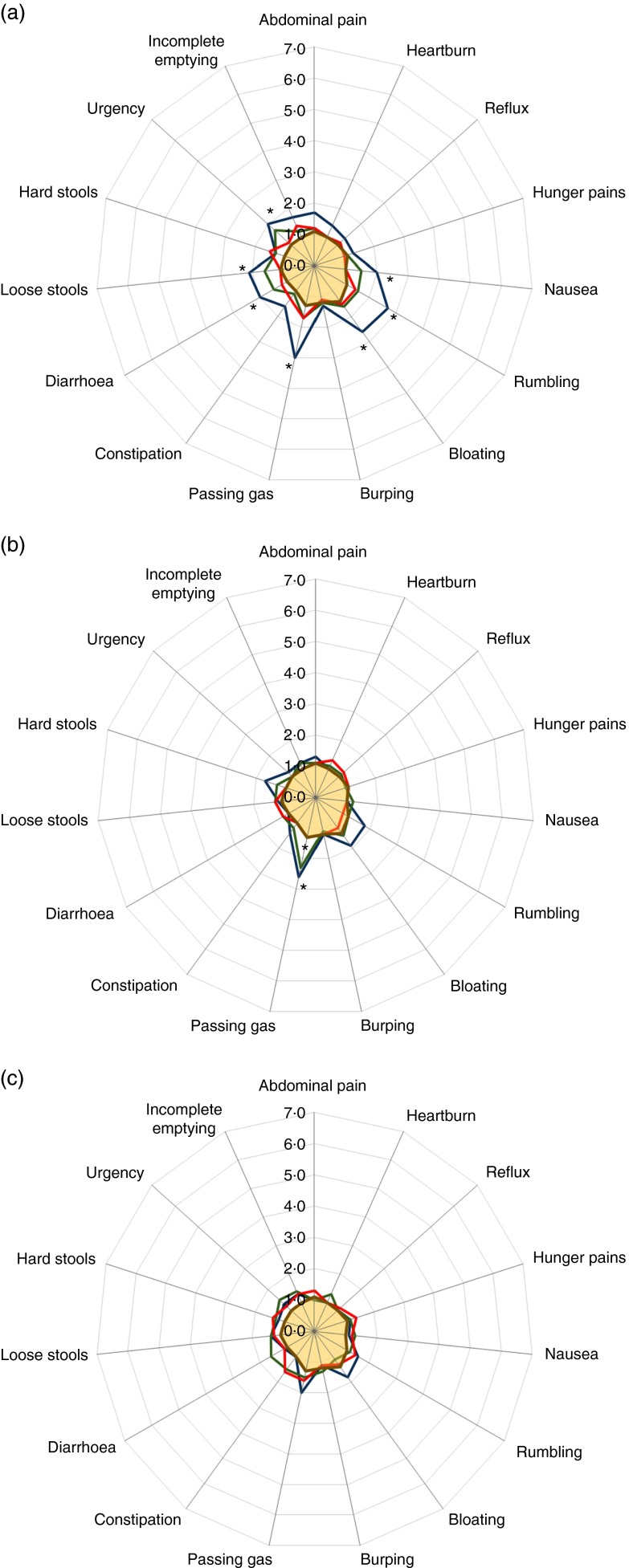
Gastrointestinal symptom rating scale (GSRS) scores at the end of the intervention. Scores ranged from 1 (no discomfort) to 7 (very severe discomfort). (a) 2′-*O*-fucosyllactose (2′FL) supplementation groups and placebo group; (b) lacto-*N*-neotetraose (LNnT) supplementation groups and placebo group; (c) 2′FL:LNnT (2:1) mix supplementation groups and placebo group. 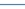, 20 g, 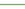, 10 g, 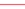, 5 g, 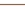, placebo. GSRS scores at the end of intervention for placebo and the intervention group were compared using a two-way ANOVA and Bonferroni’s multiple comparisons correction. * Significantly different between the intervention group and the placebo group (*P*<0·05).

GI symptoms were assessed before, during and at the end of the intervention using a self-administered GSRS questionnaire covering symptoms related to abdominal pain, indigestion, reflux, diarrhoea and constipation. The GSRS scores were low at baseline, reflecting exclusion criteria, and remained low after intervention. Compared with baseline, the changes in GSRS scores within an intervention group were generally not significant, with a few exceptions: volunteers taking the high 20-g dose of 2′FL and LNnT reported increased bloating and passing of gas. Those receiving 20 g of 2′FL further reported increased rumbling, whereas those on 20 g of LNnT reported harder stools. Increases in passing gas were also reported by those receiving 10 g of LNnT. Compared with placebo, most of the changes in GSRS scores were insignificant, again with the exception of the intervention group receiving the highest dose of 2′FL, who reported increased nausea, rumbling, bloating, passing of gas, diarrhoea, loose stools and urgency to pass stools, and the groups receiving the high 20-g dose and intermediate 10-g dose of LNnT, who reported increased passing of gas after 2 weeks of intervention (Fig. 2). Despite statistical significance, mean scores remained low (mean score<3; mild discomfort or below). No significant changes in GSRS were found in subjects receiving the highest dose of the mix.

Generally, the interventions had a minor impact on stool frequency and consistency (Table 2). The average number of daily bowel movements was significantly increased at the end of the intervention in groups taking 20 g of 2′FL, 20 g of LNnT and 5 g of LNnT compared with baseline; however, the differences were small (an extra 0·3 bowel movement/d) and deemed clinically irrelevant. When comparing the intervention groups with the placebo, no significant difference in bowel movement was observed. Subjects taking the high 20-g dose of 2′FL or LNnT reported significantly higher BSFS scores (indicating softer stools), after the intervention compared with baseline. However, the differences were small (<0·5 points increase). In addition, a number of blood and faecal biomarkers were measured, and safety of the HMO supplementation was confirmed at the level of clinical chemistry and haematology. All parameters measured remained within the normal range throughout the intervention. Although a few differences were statistically significant (online Supplementary Table S1), they were not considered to be clinically relevant. Taken together, these data demonstrated that dietary supplementation with high doses of 2′FL and LNnT is safe and well tolerated and resulted in 100 % compliance.

**Table 2:** Stool characteristics during the 2 week intervention for healthy adult volunteers (Grand mean values and standard deviations of BSFS recorded daily during the screening period (before) and during (after) the intervention)†

	2′FL						LNnT						Mix							
	20 g		10 g		5 g		20 g		10 g		5 g		20 g		10 g		5 g		Placebo	
	Mean	sd	Mean	sd	Mean	sd	Mean	sd	Mean	sd	Mean	sd	Mean	sd	Mean	sd	Mean	sd	Mean	sd
Bowel movement																				
B	1·3	0·3	1·4	0·6	1·4	0·6	1·4	0·5	1·5	0·6	1·3	0·2	1·2	0·4	1·3	0·5	1·4	0·6	1·2	0·3
A	1·6*	0·4	1·6	0·8	1·3	0·4	1·7*	0·6	1·6	0·7	1·6*	0·3	1·4	0·5	1·4	0·6	1·3	0·5	1·3	0·4
Stool consistency																				
B	3·5	0·7	3·9	0·8	3·7	0·8	3·6	0·7	3·8	0·3	4·0	0·7	3·8	0·6	3·7	0·7	4·1	0·8	3·9	0·8
A	4·0*	0·5	4·0	0·7	3·8	0·6	3·9*	0·8	3·8	0·5	4·1	0·8	3·7	0·9	4·0	0·6	4·5	0·9	3·9	0·8

2′FL, 2′-*O*-fucosyllactose; LNnT, lacto-*N*-neotetraose; Mix, 2′FL:LNnT (2:1); B, before; A, after. * The difference in stool characteristics from before and after intervention for each individual group was determined using Wilcoxon’s signed-rank test (*P*<0·05).

† Bowel movement: number of daily bowel movements. Stool consistency: measured with the Bristol Stool Form Scale (1=hard lumps; 7=liquid stools).

### Microbiota profiling and bacterial metabolites

Sequencing of the 16S rRNA V3–V4 regions yielded 123 283 020 paired-end reads, which resulted in 19 718 714 sequences after quality filtration and chimera removal distributed over 400 samples. Taxonomic assignment of OTU was done, and after this samples were rarified to the lowest sequence number found in a sample, which was 11 568 sequences. The two samples collected before the intervention were counted as one group, assigned before. The two samples collected at 1 and 2 weeks after intervention were also counted as one group, assigned after.

**Fig. 3:**
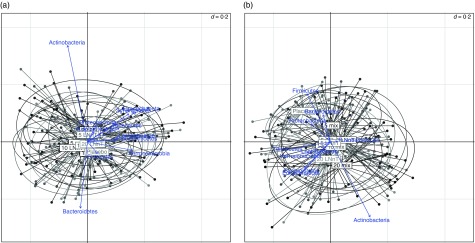
Principal coordinates analysis plot of generalised UniFrac distances for all samples collected. (a) Before intervention and (b) after intervention. Phyla abundances are overlaid in blue. Samples are divided into intervention groups with the label at the centre of gravity for each group. Before intervention, there is no clear pattern. After intervention, the human milk oligosaccharide supplementation groups followed an axis of increasing Actinobacteria and decreasing Firmicutes for increasing doses.

**Fig. 4:**
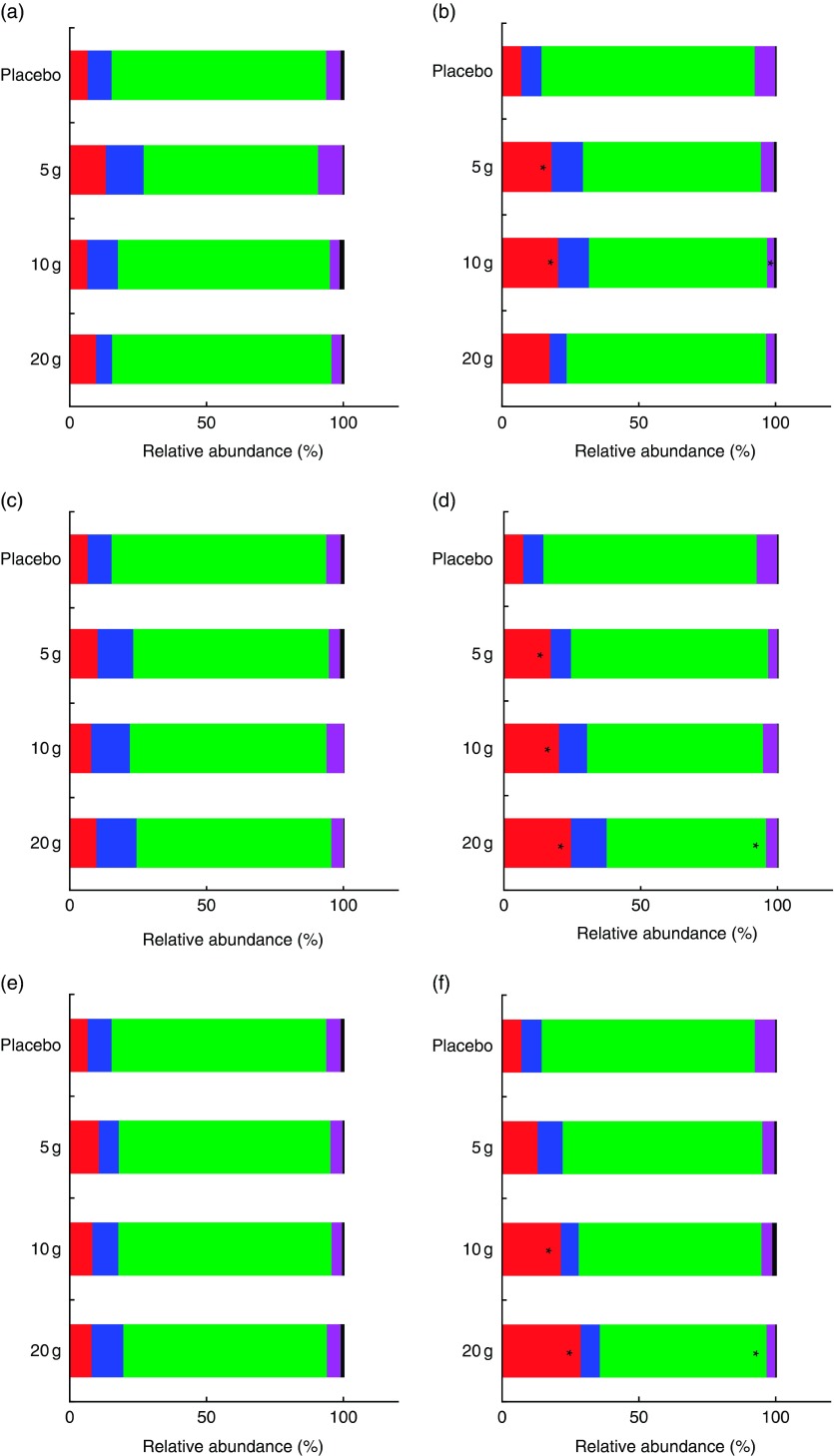
Relative abundance of faecal bacteria at the phylum level. (a and b) Phyla level in the three 2′-*O*-fucosyllactose (2′FL) groups receiving 5, 10 or 20 g and placebo before and after intervention; (c and d) phyla level in the three lacto-*N*-neotetraose (LNnT) groups receiving 5, 10 or 20 g and placebo before and after intervention; (e and f) phyla level in the three mix groups receiving 5, 10 or 20 g of 2′FL:LNnT (2:1) and placebo before and after intervention. * Significantly different between before and after intervention (*P*<0·05). 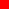, Actinobacteria; 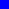, Bacteroidetes; 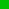, Firmicutes; 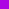, Proteobacteria; 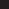, others.

Before the intervention, a plot of the UniFrac distances showed no easily discernible pattern to discriminate between the different intervention groups (Fig. 3(a)). However, after the intervention (Fig. 3(b)), the groups receiving HMO appeared to be differentiated from placebo based on the abundance of Actinobacteria, with the intervention groups receiving higher doses of HMO having greater sequence abundance of Actinobacteria (Fig. 3). Compared with baseline, the increase in the relative abundance of Actinobacteria was statistically significant for all groups taking LNnT, the high 20-g dose and the intermediate 10-g dose of the mix and for the groups taking the low 5-g dose and the intermediate 10-g dose of 2′FL (Fig. 4). Surprisingly, this effect was not observed in those taking the high dose of 2′FL. The increase in Actinobacteria sequence abundance was dose dependent, especially when excluding 20 g 2′FL: a multiple linear regression using data from all but this treatment group found that there was a significant positive correlation between the concentration of the two HMO and the increase in Actinobacteria (*P*<0·05, *R* ^2^=28 %) with LNnT and 2′FL having similar coefficients (0·008 and 0·011, respectively).

HMO supplementation also affected other phyla such as Firmicutes, which decreased after the high dose of LNnT and the mix, and Proteobacteria, which decreased after the intermediate dose of 2′FL (Fig. 4). The HMO intervention led to reduced relative abundance of Firmicutes and Proteobacteria. This phylum includes pathobionts such as *Enterobacteriaceae*.

**Fig. 5:**
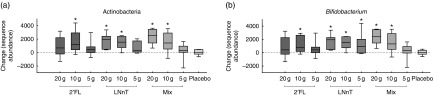
Change in sequence abundance of Actinobacteria (a) and *Bifidobacterium* (b). The box represents the median and the 25th to 75th percentiles. The whiskers represent the smallest and largest changes observed. * Significantly different between the intervention group and the placebo group (*P*<0·05). 2′FL, 2′-*O*-fucosyllactose; LNnT, lacto-*N*-neotetraose; Mix, 2′FL:LNnT (2:1).

**Fig. 6:**
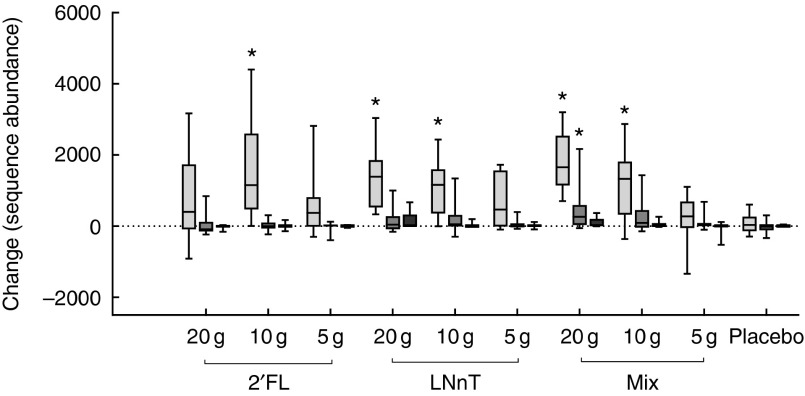
Change in sequence abundance of three operational taxonomic units (OTU) showing high similarity to the described *Bifidobacterium* species, *B. adolescentis*, *B. longum* and *B. bifidum*. The box represents the median and the 25th to 75th percentiles. The whiskers represent the smallest and largest changes observed. * Significantly different between the intervention group and the placebo group (*P*<0·05). 2′FL, 2′-*O*-fucosyllactose; LNnT, lacto-*N*-neotetraose; Mix, 2′FL:LNnT (2:1). 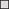, *s1_r64*; 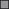, *s1_r2031*; 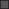, *s1_r379*.

**Fig. 7:**
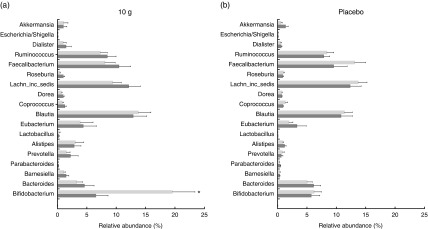
Relative abundance of faecal bacteria at the genus level from before and after intervention. The eighteen genera, *Bifidobacterium*, *Bacteroides*, *Barnesiella*, *Parabacteroides*, *Prevotella*, *Alistipes*, *Lactobacillus*, *Eubacterium*, *Blautia*, *Coprococcus*, *Dorea*, *Lachnospiracea incertae sedis*, *Roseburia*, *Faecalibacterium*, *Ruminococcus*, *Dialister*, *Escherichia/Shigella* and *Akkermansia*, selected have been associated with obesity, irritable bowel syndrome or inflammatory bowel disease. (a) The mean of relative abundance of eighteen genera from the three intervention groups given 10 g of human milk oligosaccharide (HMO). (b) Relative abundance of eighteen genera from the placebo. Values are means, with their standard errors represented by vertical bars. Multiple *t* test was performed followed by a calculation of false discovery rate indicated as an adjusted *P*-value. * Significantly different between the groups (*P*<0·05). Lachn_inc_sedis, *Lachnospiracea incertae sedis*; 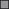, Before; 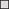, after.

**Fig. 8:**
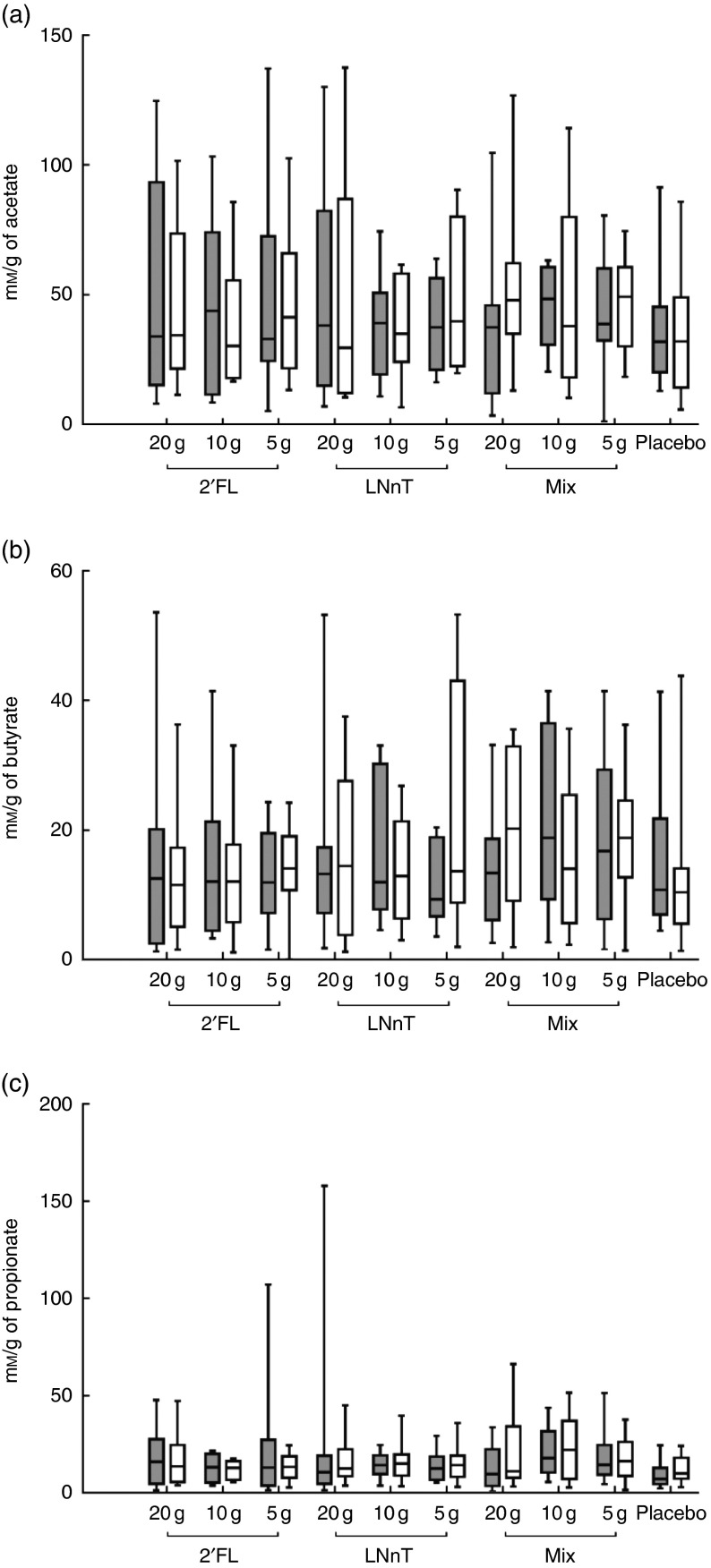
SCFA in faeces. Concentrations are given as mm/g faeces of acetate (a), butyrate (b) and propionate (c) in samples from each intervention group and placebo before (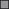) and after (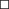) intervention. The box represents the median and the 25th to 75th percentiles. The whiskers represent the smallest and largest concentrations measured. 2′FL, 2′-*O*-fucosyllactose; LNnT, lacto-*N*-neotetraose; Mix, 2′FL:LNnT (2:1).

At the lower taxonomic level, the increase in Actinobacteria could be fully explained by the increase in *Bifidobacterium*, as this genus showed equal changes in sequence abundance as at the phylum level (Fig. 5). The *Bifidobacterium* abundance was significantly increased compared with placebo for groups taking 10 g of 2′FL, 5, 10 or 20 g of LNnT, and 10 or 20 g of the mix. In total, 77 % of the participants responded to the HMO interventions. We defined a responder as a participant having an increase in sequence abundance of *Bifidobacterium* >10 %. There was no statistically significant association, indicating that the initial abundance of *Bifidobacterium* determined whether an individual was a responder or non-responder to the bifidogenic effect of 2′FL and LNnT (Mann–Whitney test, *P*=0·359). In addition, no correlation could be observed between initial abundance and change in bifidobacteria (linear regression, *r* ^2^=0·016; *P*=0·284). Three dominant OTU (s1_r64, s1_r2031 and s1_r379) belonging to *Bifidobacterium* were affected by HMO supplementation. The changes in abundance of these OTU are shown in Fig. 6. The OTU most affected by HMO supplementation was s1_r64. The abundance of this OTU increased after HMO intervention. Compared with placebo, this change was statistically significant for groups taking 10 g of 2′FL and 10 or 20 g of LNnT or mix. The abundance of s1_r2031 increased significantly compared with placebo only for those taking 20 g of the mix. The three OTU were identified using Blastn and showed high sequence similarity to *B. adolescentis* (>99 %) for s1_r64, to *B. longum* (>99 %) for s1_r2031 and to *B. bifidum* (>99 %) for s1_r379. The effect of the HMO intervention on eighteen selected genera – *Bifidobacterium*, *Bacteroides*, *Barnesiella*, *Parabacteroides*, *Prevotella*, *Alistipes*, *Lactobacillus*, *Eubacterium*, *Blautia*, *Coprococcus*, *Dorea*, *Lachnospiracea incertae sedis*, *Roseburia*, *Faecalibacterium*, *Ruminococcus*, *Dialister*, *Escherichia/Shigella* and *Akkermansia –* associated with health or disease in obesity, irritable bowel syndrome or inflammatory bowel disease^(^ ^55^ ^–^ ^57^ ^)^ was examined. As shown in Fig. 7, 10 g of HMO did not affect the relative abundance of these genera other than *Bifidobacterium* during the 2 weeks of intervention. Similar results were observed for the other two doses – 5 and 20 g (data not shown). For the placebo group, none of the eighteen genera changed. Despite shifts in microbial composition, no significant difference in the SCFA acetate, butyrate or propionate was observed after 2 weeks of intervention (Fig. 8). Pearson’s correlation was applied to determine the relationship between bifidobacteria and SCFA concentration. A positive correlation was found between propionate and bifidobacteria in those taking 10 g of HMO (*r* 0·418; *P*<0·05). The opposite was found for subjects taking 5 g of HMO, where a negative correlation was obtained between acetate or propionate and bifidobacteria (*r* −0·357; *P*<0·05 or *r* −0·404; *P*<0·05, respectively). In breast-fed infants, *Bifidobacterium* species such as *B. longum* subsp. *infantis*, *B. breve* and *B. bifidum* dominate. In this adult study, we determined that particularly one OTU (s1_r64) with high sequence similarity to *B. adolescentis* is the main responder to 2′FL and LNnT supplementation.

## Discussion and conclusion

This study provides the first assessment on the safety, tolerance and influence on adult gut microbiota populations of 2′FL, LNnT and a mix of 2′FL and LNnT. All 100 healthy, adult participants completed the study according to the protocol, without any premature discontinuation, thus demonstrating that the daily uptake of up to 20 g of the HMO 2′FL and LNnT is perfectly safe in adults.

Blood safety assessments and physical examinations revealed no irregularities considered due to intake of the study products in any intervention group. Adverse events reported related mainly to GI symptoms, particularly gas/flatulence, and were characterised as mild. The relationship between the reported adverse events to intake of the study products was mainly described as ‘possible’. However, as many of the symptoms reported were common GI symptoms, it was difficult to judge whether the symptoms were actually related to the study product or to normal day-to-day variation or increased awareness of GI symptoms during the trial period. In all, the study raised no safety concerns. Preclinical toxicology studies of chemically produced 2′FL and LNnT have previously confirmed the safety of intake^(^ ^44^ ^,^ ^45^ ^)^, and an EFSA panel on Dietetic Products, Nutrition and Allergies (NDA) has further assessed and concluded that 2′FL and LNnT as produced by Glycom A/S are safe for use in foods^(^ ^46^ ^,^ ^47^ ^)^.

HMO as a part of breast milk are well tolerated by infants even at high doses, because the mother’s milk contains 5–25 g/l of HMO^(^ ^18^ ^)^. However, the adult tolerance to high bolus doses of selected HMO was unknown before this study. The doses used were selected from a safety and tolerance perspective and based on the average daily intake of 2′FL and LNnT in infants. The intake of 2′FL can be approximated to 170–660 mg/kg body weight per d and potentially up to 1150 mg/kg body weight per d^(^ ^46^ ^)^, and the intake of LNnT can be approximated to 20–100 mg/kg body weight per d and potentially up to 385 mg/kg body weight per d^(^ ^47^ ^)^. On a 70-kg body weight basis for adults, these values correspond to 12–46 g 2′FL per d and potentially up to 80 g per d and 1·4–7 g of LNnT per d and potentially up to 27 g LNnT per d. The maximum 20 g per daily bolus dose was selected after comparison with other prebiotic oligosaccharides that are commonly used in adult applications and that typically show pronounced tolerability issues beyond this dose^(^ ^58^ ^)^. The infant intestinal microbiota is very different from that of the adult and contains greater abundance of bacteria, particularly bifidobacteria, which are known to metabolise HMO^(^ ^37^ ^)^. Therefore, infant tolerance cannot be assumed to be a basis for adult tolerance, and therefore doses lower than the potential maximum infant exposure were selected. To assess the adult tolerance of daily boluses of 2′FL and LNnT, the participants were asked to fill in a GSRS questionnaire. At entry, the mean score on the GSRS total was less than the population norm of 1·53 based on a Swedish adult background population^(^ ^59^ ^)^. The mean scores on the GSRS total after intervention remained below the population norm except for the group receiving the 20-g dose of 2′FL. This increased to 1·87, which is still rated as minor discomfort on the GSRS scale. For individual symptoms, an increase in passing gas and bloating was observed for the higher doses of 2′FL and LNnT alone. However, even these scores remained low during the intervention and were rated as mild discomfort on the GSRS scale. Therefore, we conclude that 2′FL, LNnT and a mix of 2′FL and LNnT are well tolerated by healthy adults even at high bolus doses. Interestingly, none of the doses of the mix of 2′FL and LNnT induced GI symptoms as measured by the GSRS. The tolerance conclusions based on the GSRS scores are corroborated by the stool frequency and stool consistency results, which revealed at most small, clinically irrelevant changes.

A main objective of this study was to assess the effect of 2′FL and LNnT on the adult gut microbiota. Several studies have examined the impact of prebiotics such as galacto-oligosaccharides and fructo-oligosaccharides on the human intestinal microbiota, although most studies only monitored a few selected bacterial taxa using qPCR or fluorescence *in situ* hybridisation^(^ ^60^ ^–^ ^62^ ^)^. Only a few studies provided comprehensive, high-resolution data of the human gut microbiota through high-throughput sequencing, after prebiotic consumption^(^ ^63^ ^,^ ^64^ ^)^.

Our study showed that the uptake of 2′FL and LNnT for 2 weeks is sufficient to modulate the adult microbiota. An increase in relative abundance of bifidobacteria, to >25 % in some individuals, and a reduction in relative abundance of two phyla, Firmicutes and Proteobacteria, were observed. This modulation occurred rapidly – namely, within 1–2 weeks – and the bifidogenic effect was significant despite being on top of a normal, non-standardised complex adult diet. The observed increase in bifidobacteria was dose dependent but unrelated to the initial bifidobacteria abundance. Different results have been observed for galacto-oligosaccharides, where the greatest bifidogenic response occurred in individuals having the highest initial bifidobacteria abundance^(^ ^61^ ^)^.

Interestingly, most of the increase in bifidobacteria abundance can be explained by the increase in a specific OTU (s1_r64). This OTU has high sequence similarity (>99 %) to *B. adolescentis*, which is surprising, given that *B. adolescentis* is not known to metabolise HMO^(^ ^65^ ^)^. However, based on the 16S rRNA sequencing data, we cannot exclude at this stage that the OTU is in fact another member of *Bifidobacterium*. To assess the impact of 2′FL and LNnT supplementation on other genera relevant to human health, we specifically looked at changes in the relative abundance of eighteen genera reported to be correlated to health or disease in conditions such as obesity, irritable bowel syndrome or inflammatory bowel disease^(^ ^55^ ^–^ ^57^ ^)^. The abundance of putative beneficial taxa such as *Faecalibacterium*, *Roseburia*, *Akkermansia* or *Lactobacillus*, however, did not decrease concomitantly to the increase in bifidobacteria observed in 2′FL- and LNnT-supplemented subjects.

Bifidobacteria have for long been regarded as beneficial members of the human gut microbiota, and low levels have been reported in obese and diabetic individuals^(^ ^41^ ^,^ ^66^ ^)^, in individuals taking antibiotics^(^ ^67^ ^)^ and in patients suffering from irritable bowel syndrome or inflammatory bowel disease^(^ ^39^ ^,^ ^68^ ^)^. Safe and well-tolerated interventions, such as HMO supplementation, thus represent approaches worth considering to replenish bifidobacteria in individuals presenting low levels of these bacteria.

In conclusion, we show that 2′FL and LNnT are safe and well tolerated in healthy adults. Intriguingly, the mix of 2′FL and LNnT was better tolerated than the individual HMO when given at high doses. We further show that both 2′FL and LNnT are specific modulators of the adult microbiota with a very specific increase in bifidobacteria, particularly one OTU (s1_r64). Our results suggest that supplementing the diet with 2′FL and LNnT may be a valuable tool to restore homoeostasis in adults having an imbalanced microbiota.

## Supplementary material

For supplementary material accompanying this paper visit http://dx.doi.org/10.1017/S0007114516003354. click here to view supplementary material
