# Supplementary material for: Oral supplementation of healthy adults with 2′-O-fucosyllactose and lacto-N-neotetraose is well tolerated and shifts the intestinal microbiota
Source: Br J Nutr. 2016 Oct 10;116(8):1356–68. doi: 10.1017/S0007114516003354 (PMC5082288; doi:10.1017/S0007114516003354)
Supplement: Supplementary file 1 [file S0007114516003354sup001.pdf]

Table S1

a)

		Before			After		
**Blood biochemistry and haematology**							
**Alanine transaminase (U/L)**							
**2'FL**	20	31.00	±	14.77	34.67	±	19.27
	10	27.00	±	8.96	26.50	±	4.60
	5	25.80	±	12.44	26.10	±	8.21
**LNnT**	20	35.20	±	34.82	24.10	±	7.16
	10	31.60	±	25.44	31.00	±	13.61
	5	28.10	±	14.66	30.40	±	7.58
**mix**	20	28.70	±	10.00	30.70	±	15.03
	10	32.90	±	16.77	39.80	±	23.17
	5	31.30	±	7.83	34.70	±	10.89
**placebo**		28.70	±	14.66	27.33	±	7.58
**Albumin (g/L)**							
**2'FL**	20	40.60	±	1.84	41.30	±	2.71
	10	40.20	±	2.78	40.60	±	3.31
	5	40.67	±	2.40	41.50	±	2.51
**LNnT**	20	40.50	±	2.59	41.10	±	3.75
	10	40.90	±	3.14	41.30	±	2.98
	5	41.80	±	4.44	43.20*	±	3.58
**mix**	20	42.20	±	2.04	43.10	±	2.85
	10	39.40	±	4.17	40.40	±	3.84
	5	41.10	±	1.52	41.30	±	1.25
**placebo**		39.80	±	4.44	39.80	±	3.58
**Alkaline phosphatase (U/L)**							
**2'FL**	20	73.70	±	19.37	73.90	±	18.91
	10	60.70	±	11.49	61.60	±	12.76
	5	59.20	±	13.89	60.00	±	13.69
**LNnT**	20	60.90	±	17.48	59.70	±	17.63
	10	70.90	±	19.84	70.89	±	23.46
	5	65.80	±	14.75	65.00	±	15.30
**mix**	20	75.60	±	20.74	81.00	±	20.47
	10	73.30	±	13.93	76.00	±	15.43
	5	71.60	±	27.13	72.90	±	26.50
**placebo**		65.10	±	14.75	64.80	±	15.30
**Basophilic granulocytes (x10^9/L)**							
**2'FL**	20	0.05	±	0.02	0.05	±	0.01
	10	0.04	±	0.01	0.04	±	0.01
	5	0.04	±	0.02	0.04	±	0.01
**LNnT**	20	0.04	±	0.02	0.04	±	0.02
	10	0.06	±	0.03	0.05	±	0.02
	5	0.04	±	0.02	0.04	±	0.02
**mix**	20	0.04	±	0.02	0.04	±	0.02
	10	0.06	±	0.03	0.06	±	0.04
	5	0.04	±	0.02	0.04	±	0.02

**placebo**		0.04	±	0.02	0.04	±	0.02
**Bilirubine (µmol/L)**							
	20	9.70	±	3.71	10.70	±	8.11
**2'FL**	10	9.00	±	5.16	10.50	±	7.76
	5	9.80	±	2.30	10.30	±	3.95
	20	12.00	±	8.38	10.60	±	5.25
**LNnT**	10	8.80	±	4.29	8.67	±	2.83
	5	12.50	±	13.01	14.20	±	6.04
	20	9.30	±	2.87	9.70	±	2.79
**mix**	10	7.80	±	1.99	8.90	±	2.81
	5	9.00	±	3.89	7.60	±	3.31
**placebo**		13.70	±	13.01	10.30	±	6.04
**Eosinofilocytes (x10^9/L)**							
	20	0.23	±	0.18	0.20*	±	0.16
**2'FL**	10	0.16	±	0.11	0.16	±	0.10
	5	0.15	±	0.14	0.13*	±	0.12
	20	0.16	±	0.13	0.11	±	0.08
**LNnT**	10	0.22	±	0.15	0.21	±	0.11
	5	0.13	±	0.10	0.13	±	0.12
	20	0.19	±	0.07	0.18	±	0.11
**mix**	10	0.17	±	0.11	0.18	±	0.13
	5	0.27	±	0.20	0.24	±	0.13
**placebo**		0.14	±	0.10	0.15	±	0.12
**Erytrocytes, vol.fr (haematocrit)**							
	20	0.42	±	0.04	0.41	±	0.04
**2'FL**	10	0.43	±	0.03	0.42	±	0.03
	5	0.43	±	0.02	0.43	±	0.03
	20	0.43	±	0.03	0.41*	±	0.04
**LNnT**	10	0.45	±	0.04	0.44	±	0.03
	5	0.43	±	0.04	0.43	±	0.04
	20	0.42	±	0.04	0.42	±	0.04
**mix**	10	0.43	±	0.03	0.42	±	0.04
	5	0.44	±	0.03	0.44	±	0.03
**placebo**		0.44	±	0.04	0.43	±	0.04
**Erytrocytes (x10^{12}/L)**							
	20	4.77	±	0.40	4.67	±	0.38
**2'FL**	10	4.78	±	0.43	4.72	±	0.41
	5	4.67	±	0.49	4.68	±	0.50
	20	4.78	±	0.42	4.55	±	0.46
**LNnT**	10	4.93	±	0.49	4.87	±	0.52
	5	4.83	±	0.47	4.82	±	0.52
	20	4.70	±	0.50	4.67	±	0.49
**mix**	10	4.81	±	0.54	4.78	±	0.43
	5	4.89	±	0.52	4.87	±	0.54
**placebo**		4.81	±	0.47	4.72	±	0.52
**Glucose (mmol/L)**							

	20	5.48	±	0.37	5.06	±	0.43
**2'FL**	10	4.96	±	0.24	4.77	±	1.04
	5	4.93	±	0.58	5.36*	±	0.66
	20	4.70	±	0.41	4.36	±	0.72
**LNnT**	10	4.60	±	0.70	5.11	±	0.83
	5	5.10	±	0.48	4.87	±	0.55
	20	4.84	±	0.40	4.44	±	1.71
**mix**	10	4.77	±	0.41	4.86	±	0.86
	5	4.79	±	0.59	4.85	±	0.53
**placebo**		4.84	±	0.48	4.97	±	0.55
**Haemoglobin (mmol/L)**							
	20	8.74	±	0.96	8.56	±	0.96
**2'FL**	10	9.03	±	0.88	8.77	±	0.79
	5	8.99	±	0.59	8.96	±	0.73
	20	9.09	±	0.78	8.67*	±	0.87
**LNnT**	10	9.28	±	0.96	9.21	±	0.74
	5	9.25	±	0.80	9.21	±	0.96
	20	8.86	±	0.92	8.91	±	0.98
**mix**	10	8.90	±	0.98	8.87	±	1.01
	5	9.10	±	0.51	9.12	±	0.61
**placebo**		9.17	±	0.80	9.04	±	0.96
**Potassium (mmol/L)**							
	20	3.98	±	0.14	3.85	±	0.23
**2'FL**	10	4.08	±	0.28	3.94	±	0.20
	5	3.98	±	0.38	3.75	±	0.25
	20	3.95	±	0.28	3.69*	±	0.15
**LNnT**	10	4.01	±	0.17	3.90	±	0.25
	5	4.02	±	0.20	3.79*	±	0.18
	20	4.04	±	0.20	3.85*	±	0.22
**mix**	10	3.97	±	0.24	3.76*	±	0.23
	5	3.94	±	0.26	3.88	±	0.33
**placebo**		3.99	±	0.20	3.87*	±	0.18
**Coagulation factors II+VII+X (%)[$]**							
	20	1.01	±	0.25	0.99	±	0.25
**2'FL**	10	0.82	±	0.21	0.88	±	0.21
	5	0.79	±	0.11	0.78	±	0.11
	20	0.88	±	0.18	0.91	±	0.18
**LNnT**	10	0.81	±	0.13	0.87	±	0.15
	5	0.90	±	0.18	0.90	±	0.18
	20	0.72	±	0.15	0.84*	±	0.24
**mix**	10	0.88	±	0.15	0.82	±	0.31
	5	0.89	±	0.21	0.96	±	0.17
**placebo**		0.90	±	0.18	0.96	±	0.18
**Creatinin (µmol/L)**							
	20	76.60	±	9.12	77.50	±	11.29
**2'FL**	10	81.20	±	20.79	75.60	±	14.28

	5	69.80	±	10.94	70.40	±	13.76
**LNnT**	20	72.70	±	12.10	73.90	±	13.48
	10	74.90	±	10.57	73.20	±	9.10
	5	85.90	±	17.12	82.80	±	19.18
**mix**	20	84.20	±	13.68	82.60	±	15.59
	10	74.70	±	16.02	76.30	±	17.67
	5	72.50	±	15.90	72.40	±	14.83
**placebo**		85.50	±	17.12	79.40	±	19.18
**Leukocytes ($x10^9/L$)**							
**2'FL**	20	7.99	±	2.67	7.16	±	2.63
	10	6.75	±	1.30	6.51	±	1.40
	5	7.26	±	2.53	7.41	±	2.30
**LNnT**	20	6.94	±	1.68	6.23	±	2.11
	10	7.50	±	3.40	6.65	±	2.16
	5	6.61	±	1.17	6.14	±	1.53
**mix**	20	6.46	±	1.52	6.22	±	1.81
	10	7.04	±	2.54	6.99	±	2.23
	5	6.87	±	1.86	6.17*	±	1.77
**placebo**		6.63	±	1.17	6.29	±	1.53
**Lymfocytes ($x10^9/L$)**							
**2'FL**	20	2.27	±	0.82	2.18	±	0.83
	10	2.11	±	0.38	1.94*	±	0.32
	5	2.19	±	0.54	2.09	±	0.44
**LNnT**	20	2.38	±	0.68	2.13	±	0.69
	10	2.16	±	0.70	1.88	±	0.73
	5	1.96	±	0.44	1.98	±	0.52
**mix**	20	2.27	±	0.62	2.04	±	0.57
	10	2.20	±	0.74	2.13	±	0.59
	5	2.29	±	0.59	2.04	±	0.61
**placebo**		2.05	±	0.44	1.93	±	0.52
**Metamyelo+Myelo+Promyelocytes ($x10^9/L$)**							
**2'FL**	20	0.03	±	0.04	0.02	±	0.03
	10	0.02	±	0.03	0.02	±	0.02
	5	0.02	±	0.04	0.03	±	0.03
**LNnT**	20	0.00	±	0.01	0.01	±	0.02
	10	0.02	±	0.04	0.02	±	0.03
	5	0.01	±	0.01	0.01	±	0.01
**mix**	20	0.00	±	0.00	0.01	±	0.01
	10	0.03	±	0.04	0.02	±	0.03
	5	0.02	±	0.03	0.01	±	0.01
**placebo**		0.00	±	0.01	0.01*	±	0.01
**Monocytes ($x10^9/L$)**							
**2'FL**	20	0.66	±	0.27	0.57	±	0.22
	10	0.47	±	0.15	0.52	±	0.15
	5	0.55	±	0.17	0.52	±	0.17
	20	0.58	±	0.12	0.47*	±	0.14

**LNnT**	10	0.62	±	0.25	0.54	±	0.26
	5	0.59	±	0.20	0.51*	±	0.26
**mix**	20	0.54	±	0.16	0.51	±	0.17
	10	0.60	±	0.19	0.53*	±	0.16
	5	0.55	±	0.13	0.47*	±	0.10
**placebo**		0.54	±	0.20	0.56	±	0.26
**Sodium (mmol/L)**							
**2'FL**	20	139.80	±	1.03	139.40	±	0.97
	10	140.10	±	1.66	139.60	±	1.71
	5	140.40	±	0.97	139.00	±	1.49
**LNnT**	20	140.40	±	0.84	140.20	±	0.92
	10	141.00	±	0.82	139.60*	±	1.26
	5	140.30	±	1.93	139.40*	±	1.34
**mix**	20	140.00	±	1.15	140.00	±	1.33
	10	139.80	±	1.93	139.40	±	1.17
	5	141.30	±	1.49	140.10	±	1.10
**placebo**		140.20	±	1.93	140.70	±	1.34
**Neutrophil granulocytes (x10^9/L)**							
**2'FL**	20	4.72	±	1.59	4.16	±	1.72
	10	3.90	±	1.11	3.84	±	1.34
	5	4.30	±	2.12	4.61	±	1.95
**LNnT**	20	3.75	±	1.13	3.45	±	1.56
	10	4.41	±	2.60	3.95	±	1.65
	5	3.85	±	0.82	3.48	±	1.22
**mix**	20	3.40	±	1.00	3.44	±	1.26
	10	3.98	±	1.73	4.05	±	1.91
	5	3.70	±	1.43	3.35	±	1.32
**placebo**		3.85	±	0.82	3.61	±	1.22
**Thrombocytes (x10^9/L)**							
**2'FL**	20	272.40	±	65.08	269.10	±	64.14
	10	259.80	±	97.38	273.30	±	101.61
	5	246.70	±	56.79	247.60	±	40.91
**LNnT**	20	234.10	±	51.45	232.20	±	49.75
	10	242.90	±	61.90	228.30	±	52.40
	5	238.20	±	74.96	246.50	±	63.36
**mix**	20	231.70	±	28.71	237.30	±	22.19
	10	260.30	±	65.01	255.20	±	45.89
	5	249.70	±	36.70	266.10	±	49.03
**placebo**		235.20	±	74.96	242.00	±	63.36

$^{\$}$ *Coagulation factors are arbitrarily set at 100%*

b) In many cases CRP were not given as a specific value. This was the case if CRP was <2.9. Hence a total mean of all subjects cannot be calculated. CRP is therfore indicated as normal or without normal ranges.

**C-reactive protein (CRP; mg/L)**

		normal	*elevated*	*normal*	*elevated*
**2'FL**	20	9	1	10	0
	10	10	0	9	1
	5	10	0	8	1
**LNnT**	20	9	1	10	0
	10	10	0	9	0
	5	10	0	10	0
**mix**	20	10	0	10	0
	10	7	3	10	0
	5	10	0	10	0
**placebo**		9	1	9	1

Frequency of normal (CRP<8.0) and elevated CRP (CRP ≥ 8) before and after supplementation.

c) In many cases GFR were not given as a specific value. This was the case if GFR was > 90. Hence a total mean of all subjects cannot be calculated. GFR is therfore indicated as normal or without normal ranges.

Glomerulary filtration rate (GFR; mL/min/1.73m^2)					
		normal	*impaired*	*normal*	*impaired*
**2'FL**	20	10	0	9	1
	10	10	0	10	0
	5	10	0	9	1
**LNnT**	20	10	0	10	0
	10	10	0	10	0
	5	10	0	10	0
**mix**	20	9	1	9	1
	10	10	0	10	0
	5	10	0	10	0
**placebo**		10	0	10	0

Frequency of normal (GFR ≥ 60) and impaired GFR (GFR<60) before and after supplementation.

d)

Blood biomarkers								
**Apolipoprotein A1 (g/L)**								
**2'FL**	20	1.69	±	0.38	1.61	±	0.35
	10	1.55	±	0.29	1.60	±	0.34
	5	1.67	±	0.25	1.55	±	0.27
**LNnT**	20	1.71	±	0.28	1.58	±	0.27
	10	1.53	±	0.23	1.56	±	0.22
	5	1.58	±	0.21	1.60	±	0.20
**mix**	20	1.57	±	0.32	1.53	±	0.27
	10	1.62	±	0.26	1.50	±	0.29
	5	1.59	±	0.24	1.69	±	0.23
**placebo**		1.68	±	0.34	1.67	±	0.32

**Apolipoprotein B (g/L)**							
**2'FL**	20	1.03	±	0.28	0.98	±	0.21
	10	0.77	±	0.17	0.75	±	0.19
	5	0.88	±	0.20	0.80	±	0.19
**LNnT**	20	0.82	±	0.26	0.77	±	0.23
	10	0.80	±	0.24	0.82	±	0.21
	5	0.85	±	0.19	0.87	±	0.18
**mix**	20	0.68	±	0.22	0.69	±	0.13
	10	0.97	±	0.25	0.98	±	0.33
	5	0.78	±	0.27	0.84	±	0.28
**placebo**		0.75	±	0.12	0.77	±	0.13
**Chol+ester in LDL (mmol/L)**							
**2'FL**	20	3.71	±	1.00	3.52	±	0.81
	10	2.59	±	0.74	2.58	±	0.70
	5	3.11	±	0.86	2.86	±	0.84
**LNnT**	20	2.79	±	1.06	2.81	±	0.94
	10	2.71	±	0.96	2.85	±	0.92
	5	2.95	±	0.64	3.04	±	0.70
**mix**	20	2.22	±	0.88	2.25	±	0.58
	10	3.23	±	0.87	3.45	±	1.13
	5	2.63	±	1.01	2.93*	±	1.08
**placebo**		2.45	±	0.60	2.64	±	0.60
**Chol+ester in HDL (mmol/L)**							
**2'FL**	20	1.42	±	0.44	1.36	±	0.38
	10	1.46	±	0.42	1.49	±	0.45
	5	1.51	±	0.39	1.41	±	0.41
**LNnT**	20	1.46	±	0.25	1.42	±	0.30
	10	1.35	±	0.28	1.33	±	0.25
	5	1.37	±	0.23	1.39	±	0.29
**mix**	20	1.43	±	0.48	1.42	±	0.43
	10	1.45	±	0.34	1.30*	±	0.33
	5	1.39	±	0.28	1.43	±	0.26
**placebo**		1.46	±	0.43	1.49	±	0.42
**Cortisol (Total) (µmol/L)**							
**2'FL**	20	230.90	±	50.37	261.40	±	124.27
	10	241.30	±	72.67	283.60	±	116.01
	5	254.00	±	110.87	258.10	±	49.63
**LNnT**	20	254.00	±	139.54	271.50	±	150.30
	10	311.40	±	125.84	345.80	±	111.93
	5	270.40	±	56.47	273.30	±	48.95
**mix**	20	290.70	±	90.36	280.80	±	65.21
	10	292.00	±	138.82	260.60	±	125.58
	5	253.00	±	125.02	263.30	±	56.72
**placebo**		317.20	±	166.39	287.00	±	155.16
**Estradiol (nmol/L)**							
	20	0.22	±	0.09	0.36	±	0.35

**2'FL**	10	0.21	±	0.13	0.43	±	0.41
	5	0.30	±	0.24	0.38	±	0.57
**LNnT**	20	0.26	±	0.19	0.25	±	0.17
	10	0.23	±	0.06	0.42*	±	0.40
	5	0.21	±	0.12	0.15	±	0.04
**mix**	20	0.16	±	0.06	0.16	±	0.05
	10	0.22	±	0.08	0.32	±	0.32
	5	0.28	±	0.33	0.16	±	0.09
**placebo**		0.32	±	0.28	0.18	±	0.06
**FFA(C6-C20) (nmol/L)**							
**2'FL**	20	0.34	±	0.24	0.35	±	0.14
	10	0.30	±	0.16	0.30	±	0.19
	5	0.35	±	0.23	0.32	±	0.17
**LNnT**	20	0.34	±	0.20	0.29	±	0.29
	10	0.33	±	0.24	0.35	±	0.17
	5	0.19	±	0.10	0.30	±	0.14
**mix**	20	0.22	±	0.17	0.20	±	0.13
	10	0.32	±	0.25	0.30	±	0.28
	5	0.32	±	0.22	0.33	±	0.18
**placebo**		0.29	±	0.25	0.28	±	0.18
**Glucagon (ng/L)**							
**2'FL**	20	119.40	±	82.59	144.00	±	80.39
	10	113.60	±	65.93	147.70*	±	68.84
	5	90.10	±	63.89	141.90*	±	55.71
**LNnT**	20	110.50	±	67.13	141.50	±	52.23
	10	114.20	±	60.86	139.50	±	61.58
	5	110.80	±	75.59	133.00	±	72.04
**mix**	20	126.60	±	72.21	145.60	±	58.87
	10	124.70	±	68.66	148.10	±	63.59
	5	114.10	±	54.35	138.00	±	54.09
**placebo**		115.90	±	70.16	156.00*	±	67.98
**Hemoglobin A1c (mmol/mol)**							
**2'FL**	20	34.50	±	3.17	34.30	±	3.16
	10	32.40	±	2.55	31.78	±	2.39
	5	33.80	±	2.44	33.10*	±	2.47
**LNnT**	20	30.40	±	1.96	30.00	±	1.83
	10	33.70	±	2.11	32.70*	±	2.45
	5	32.70	±	2.45	32.90	±	2.69
**mix**	20	32.80	±	3.79	32.60	±	3.98
	10	34.40	±	3.60	34.70	±	4.00
	5	34.30	±	3.37	33.50*	±	3.41
**placebo**		31.20	±	2.15	30.80	±	2.20
**Insulin (nmol/L)**							
**2'FL**	20	88.90	±	168.91	107.38	±	80.97
	10	44.44	±	26.72	40.60	±	29.08
	5	70.50	±	36.98	102.00	±	94.42

**LNnT**	20	76.56	±	71.64	57.44	±	36.94
	10	111.30	±	101.07	96.40	±	81.14
	5	49.60	±	34.27	67.40	±	46.76
**mix**	20	89.00	±	69.52	89.22	±	54.52
	10	147.80	±	140.22	75.11*	±	50.51
	5	81.20	±	69.20	66.22	±	39.45
**placebo**		60.00	±	44.98	84.30	±	62.33
**Iron (µmol/L)**							
**2'FL**	20	18.78	±	9.64	16.78	±	9.61
	10	15.10	±	6.40	14.00	±	6.62
	5	18.50	±	5.44	14.50	±	5.06
**LNnT**	20	13.80	±	4.34	11.60	±	3.57
	10	18.50	±	6.24	21.50	±	5.30
	5	18.30	±	3.23	18.90	±	5.97
**mix**	20	19.40	±	5.21	16.00	±	4.81
	10	14.50	±	7.18	16.80	±	6.51
	5	18.90	±	3.48	16.30	±	4.14
**placebo**		16.90	±	8.62	16.00	±	7.62
**Lysozyme (nmol/L)**							
**2'FL**	20	1000.20	±	506.16	1108.67	±	403.04
	10	686.30	±	132.35	872.20*	±	239.10
	5	747.30	±	181.84	982.00*	±	318.67
**LNnT**	20	708.60	±	247.86	694.00	±	201.56
	10	895.00	±	175.20	1034.20	±	325.54
	5	851.30	±	93.60	953.80	±	164.74
**mix**	20	857.20	±	251.18	920.60	±	351.51
	10	828.10	±	365.53	970.67	±	240.95
	5	832.90	±	221.62	803.80	±	217.57
**placebo**		854.20	±	185.05	946.11	±	216.50
**Progesterone (nmol/L)**							
**2'FL**	20	3.37	±	4.72	4.42	±	4.99
	10	4.01	±	5.78	3.26	±	4.34
	5	8.45	±	16.52	4.67	±	5.60
**LNnT**	20	7.18	±	13.47	7.22	±	9.75
	10	1.56	±	0.95	4.75	±	6.49
	5	5.21	±	11.64	1.04	±	0.27
**mix**	20	0.93	±	0.21	1.16	±	0.46
	10	1.70	±	0.71	1.42	±	0.68
	5	4.27	±	8.26	1.37	±	0.46
**placebo**		14.38	±	19.37	0.97	±	0.38
**Total testosterone (nmol/L)**							
**2'FL**	20	11.45	±	13.64	7.68*	±	11.32
	10	10.81	±	15.42	9.78	±	12.73
	5	6.52	±	7.97	6.48	±	8.56
**LNnT**	20	11.05	±	13.44	9.55	±	11.33
	10	9.50	±	11.88	9.88	±	12.03

	5	18.35	±	9.42	15.86*	±	7.49
mix	20	16.40	±	8.95	14.79	±	8.48
	10	6.15	±	7.65	6.94	±	8.30
	5	10.07	±	9.27	12.08	±	10.25
**placebo**		6.44	±	9.83	5.18	±	7.30
			**Transferrin (g/L)**				
**2'FL**	20	2.71	±	0.70	2.70	±	0.66
	10	2.27	±	0.56	2.38	±	0.65
	5	2.55	±	0.28	2.44	±	0.39
**LNnT**	20	2.52	±	0.49	2.50	±	0.66
	10	2.43	±	0.29	2.44	±	0.24
	5	2.42	±	0.30	2.47	±	0.31
**mix**	20	2.37	±	0.37	2.38	±	0.22
	10	2.64	±	0.25	2.64	±	0.43
	5	2.46	±	0.42	2.62	±	0.46
**placebo**		2.56	±	0.52	2.66	±	0.48
			**Triglyceride (mmol/L)**				
**2'FL**	20	2.50	±	2.74	2.21	±	2.14
	10	1.20	±	0.80	1.08	±	0.81
	5	1.53	±	1.09	1.13	±	0.41
**LNnT**	20	1.26	±	0.74	1.11	±	0.66
	10	1.34	±	0.91	1.27	±	0.55
	5	1.35	±	0.78	1.56	±	0.84
**mix**	20	1.05	±	0.45	1.13	±	0.72
	10	2.09	±	1.60	2.11	±	1.43
	5	1.37	±	1.05	1.61	±	0.68
**placebo**		0.96	±	0.45	1.33	±	0.94
			**Blood Urea Nitrogen (BUN) (mmol/L)**				
**2'FL**	20	5.44	±	1.63	5.39	±	0.78
	10	5.12	±	1.16	5.04	±	1.13
	5	4.66	±	0.86	4.47	±	1.20
**LNnT**	20	5.19	±	1.47	5.01	±	1.50
	10	4.36	±	1.09	4.62	±	0.67
	5	5.69	±	1.17	5.67	±	1.19
**mix**	20	5.34	±	0.89	5.56	±	0.98
	10	5.09	±	1.31	5.07	±	1.16
	5	5.29	±	1.19	5.04	±	1.12
**placebo**		4.63	±	1.08	4.92	±	2.17

e) In many cases the cytokines were not given as a specific value due to levels below the method detection limit. Hence a total mean of all subjects cannot be calculated. The cytokines are therfore indicated as normal or without normal ranges.

		**Interleukin-10 (pg/mL)**			
		normal	***elevated***	***normal***	***elevated***
**2'FL**	20	10	0	10	0
	10	10	0	10	0

	5	10	0	10	0
	20	10	0	10	0
**LNnT**	10	8	2	9	1
	5	8	2	8	2
	20	10	0	10	0
**mix**	10	9	1	9	1
	5	8	2	8	2
**placebo**		10	0	10	0

Method detection limit: <5 pg/mL
Normal range (0-9.1 pg/mL) and elevated (>9.1 pg/mL) before and after supplementation.

**Interleukin-6 (pg/mL)**					
		normal	***elevated***	***normal***	***elevated***
	20	10	0	10	0
**2'FL**	10	10	0	10	0
	5	10	0	10	0
	20	10	0	10	0
**LNnT**	10	10	0	10	0
	5	10	0	10	0
	20	10	0	10	0
**mix**	10	10	0	10	0
	5	10	0	10	0
**placebo**		9	1	10	0

Method detection limit: <2 pg/mL
Normal range (0-5.9 pg/mL) and elevated (>5.9 pg/mL) before and

**TNF-alfa (pg/mL)**					
		normal	***elevated***	***normal***	***elevated***
	20	10	0	10	0
**2'FL**	10	10	0	10	0
	5	10	0	10	0
	20	10	0	10	0
**LNnT**	10	10	0	10	0
	5	10	0	10	0
	20	10	0	10	0
**mix**	10	10	0	10	0
	5	10	0	10	0
**placebo**		10	0	10	0

Method detection limit: <15.6 pg/mL
Normal range (0-15.6 pg/mL) and elevated (>15.6 pg/mL) before

f)

Faecal biomarkers

**Calprotectin (µg/g)**							
	20	46.42	±	32.41	39.67	±	55.42
**2'FL**	10	36.98	±	22.52	40.78	±	56.57
	5	66.18	±	99.80	26.52	±	23.75
	20	49.32	±	42.11	24.24	±	23.38

**LNnT**	10	27.94	±	22.89	35.34	±	34.54
	5	31.95	±	38.09	24.94	±	15.28
**mix**	20	51.43	±	58.78	50.91	±	76.26
	10	27.57	±	22.88	23.72	±	15.92
	5	49.82	±	53.93	32.22	±	14.62
**placebo**		20.17	±	11.33	41.19	±	40.23
**IgA (μg/mL)**							
**2'FL**	20	967.37	±	932.09	739.71	±	844.39
	10	1076.32	±	1007.04	1240.98	±	1252.26
	5	1467.84	±	1275.02	938.38	±	720.12
**LNnT**	20	1966.20	±	1714.82	707.65*	±	498.63
	10	1641.80	±	848.33	1042.50	±	977.89
	5	649.01	±	675.64	1203.45	±	718.90
**mix**	20	730.73	±	647.20	1427.27	±	1651.86
	10	606.29	±	475.67	815.38	±	722.32
	5	1099.97	±	736.93	1391.97	±	1319.12
**placebo**		784.05	±	728.34	519.33	±	393.79

Values are mean±SD of blood safety and faecal biomarker concentration measured at entry and end of the intervention. *indicates significant difference in concentration measured at entry and end of intervention for each individual group determind using Wilcoxon signed-rank test ($p<0.05$).
